# Changes in mortality rates and causes of death in a population-based cohort of persons living with and without HIV from 1996 to 2012

**DOI:** 10.1186/s12879-017-2254-7

**Published:** 2017-02-27

**Authors:** Oghenowede Eyawo, Conrado Franco-Villalobos, Mark W. Hull, Adriana Nohpal, Hasina Samji, Paul Sereda, Viviane D. Lima, Jeannie Shoveller, David Moore, Julio S. G. Montaner, Robert S. Hogg, for the Comparative Outcomes And Service Utilization Trends (COAST) study, Julio Montaner, Robert Hogg, Oghenowede Eyawo, Mark Hull, Jeannie Shoveller, David Moore, Paul Sereda, Viviane Lima

**Affiliations:** 1 British Columbia Centre for Excellence in HIV/AIDS, St. Paul’s Hospital, 608-1081 Burrard Street, Vancouver, BC Canada; 2 Faculty of Health Sciences, Simon Fraser University, Burnaby, BC Canada; 3 DataClever Consulting Inc., Vancouver, BC Canada; 4 British Columbia Centre for Disease Control, Vancouver, BC Canada; 5 School of Population and Public Health, University of British Columbia, Vancouver, BC Canada; 6 Department of Medicine, University of British Columbia, Vancouver, BC Canada

**Keywords:** Mortality, HIV infection, Age-standardized mortality rate, Mortality rate ratio, Cause of death

## Abstract

**Background:**

Non-HIV/AIDS-related diseases are gaining prominence as important causes of morbidity and mortality among people living with HIV. The purpose of this study was to characterize and compare changes over time in mortality rates and causes of death among a population-based cohort of persons living with and without HIV in British Columbia (BC), Canada.

**Methods:**

We analysed data from the Comparative Outcomes And Service Utilization Trends (COAST) study; a retrospective population-based study created via linkage between the BC Centre for Excellence in HIV/AIDS and Population Data BC, and containing data for HIV-infected individuals and the general population of BC, respectively. Our analysis included all known HIV-infected adults (≥ 20 years) in BC and a random 10% sample of uninfected BC adults followed from 1996 to 2012. Deaths were identified through Population Data BC – which contains information on all registered deaths in BC (BC Vital Statistics Agency dataset) and classified into cause of death categories using International Classification of Diseases (ICD) 9/10 codes. Age-standardized mortality rates (ASMR) and mortality rate ratios were calculated. Trend test were performed.

**Results:**

3401 (25%), and 47,647 (9%) individuals died during the 5,620,150 person-years of follow-up among 13,729 HIV-infected and 510,313 uninfected individuals, respectively. All-cause and cause-specific mortality rates were consistently higher among HIV-infected compared to HIV-negative individuals, except for neurological disorders. All-cause ASMR decreased from 126.75 (95% CI: 84.92-168.57) per 1000 population in 1996 to 21.29 (95% CI: 17.79-24.79) in 2011-2012 (83% decline; *p* < 0.001 for trend), compared to a change from 7.97 (95% CI: 7.61-8.33) to 6.87 (95% CI: 6.70-7.04) among uninfected individuals (14% decline; *p* < 0.001). Mortality rates from HIV/AIDS-related causes decreased by 94% from 103.85 per 1000 population in 1996 to 6.72 by the 2011–2012 era (*p* < 0.001). Significant ASMR reductions were also observed for hepatic/liver disease and drug abuse/overdose deaths. ASMRs for neurological disorders increased significantly over time. Non-AIDS-defining cancers are currently the leading non-HIV/AIDS-related cause of death in both HIV-infected and uninfected individuals.

**Conclusions:**

Despite the significant mortality rate reductions observed among HIV-infected individuals from 1996 to 2012, they still have excess mortality risk compared to uninfected individuals. Additional efforts are needed to promote effective risk factor management and appropriate screening measures among people living with HIV.

## Background

The widespread uptake of combination antiretroviral therapy (cART) has led to substantial reductions in morbidity and mortality associated with HIV/AIDS [1–3]. Over the last 20 years, cART regimens have become, not only more effective and less toxic, but also simpler in terms of pill burden and frequency, thus enhancing adherence [4, 5]. This has translated into improvement in survival among cART-treated persons living with HIV [6]. Today in many regions, HIV/AIDS is widely viewed as a manageable chronic condition [7], with the life expectancy of HIV-infected individuals receiving cART approaching that of the general population in some settings [8–10].

Improved survival rates now observed among HIV-infected individuals treated with cART have been accompanied by a gradual shift in their morbidity and mortality patterns in some studies [11, 12]. Non-HIV-specific diseases, including non-AIDS-defining cancers, cardiovascular diseases (CVD), renal and liver diseases, are becoming more prevalent with many HIV-infected individuals now experiencing one or more of these comorbid conditions [11–16]. As a result, causes of death among people living with HIV have shifted in several important ways. In particular, deaths from these chronic diseases and other complications typically associated with natural aging have gained prominence as individuals are living longer on cART. It is thus important to monitor changes in the causes of death as this information will be useful in projecting future morbidity and mortality trends. Ultimately, such knowledge will provide guidance that may inform how risk factors for such increasingly common adverse health outcomes are addressed in this population.

Although decreases in all-cause mortality rates have been well documented among HIV-infected individuals in British Columbia (BC), Canada [1, 6, 17–21], changes in cause-specific deaths in this population following cART introduction in this setting is less well characterized. Furthermore, it is unclear how the trends and causes of death among HIV-infected individuals in this setting compare to that of the general uninfected population. Given the changing pattern of causes of death reported among HIV-infected individuals in other settings after cART introduction [11–14], we hypothesized that similar patterns may be observed among HIV-infected BC residents. Our objective was, therefore, to characterize the changes in mortality rates and causes of death over time following cART introduction among a population-based cohort of HIV-infected individuals in BC. Secondly, we compared these patterns of death to that observed in a population-based sample of uninfected individuals drawn from the BC general population over the same time period.

## Methods

### Study population and setting

We used data from the Comparative Outcomes And Service Utilization Trends (COAST) study. COAST is a large population-based retrospective cohort study including longitudinal data of HIV-infected adults (≥ 19 years) and a control group from the general population of BC residents meeting the age criterion and recruited into the cohort in the period between April 1, 1996 and March 31, 2013. Briefly, the COAST cohort was designed to characterize health outcomes and healthcare utilization of persons living with HIV infection since the introduction of cART and to evaluate differences in these parameters from those observed in the general population. COAST contains de-identified health-related data arising from a unique data linkage between the BC Centre for Excellence in HIV/AIDS (BCCfE) Drug Treatment Program [22] and Population Data BC [23]. The Drug Treatment Program manages the province-wide antiretroviral therapy dispensation program. It prospectively collects demographic, immunologic, virologic, ART-use and other clinical data on all known HIV-infected individuals who have ever accessed ART, which is accessible at no cost through the BCCfE. Population Data BC is BC’s repository of individual-level longitudinal data from health administrative databases [24–31]; data that are collected by public bodies for all four million BC residents.

The follow-up for this analysis started at cohort entry and ended in December 31, 2012. The current analysis is limited to COAST study participants aged 20 years or older with at least one day of follow-up since entering the cohort. This age criterion was necessary to facilitate age groupings by five-year categories and thus permit age standardization of mortality rates. The study population comprised of two distinct cohorts distinguished by their HIV status. The HIV-infected cohort is comprised of all age-eligible individuals known to be HIV-positive during the follow-up period. HIV diagnosis was based on the presence of at least one detectable HIV plasma viral load, and/or an initiation of ART as indicated in the BCCfE Drug Treatment Program registry. The HIV case identification was supplemented through administrative health records review of International Classification of Diseases (ICD) – 9 and 10 codes for records with at least one documentation of having received care for an HIV/AIDS-related medical condition or death. The following HIV-related ICD 9/10 codes were used: (i) ICD-9 codes 042–44, V08, 795.71, 795.8; (ii) ICD-10 codes B20-24, R75, Z21; and (iii) ICD-10-CA codes B20-24, R75, Z21. To avoid potential misclassification, we applied a validated HIV case-finding algorithm – an additional criteria of ≥ 1 inpatient and/or ≥ 3 outpatient ICD-9/10 codes to potential cases identified within administrative health records [32]. The uninfected cohort was created based on a ten percent randomly generated sample of adult individuals from the general population of BC over the same time frame and excludes any known HIV-infected individual. The random sample in COAST was created by executing a computer-generated simple random sampling technique to draw a ten percent sample from a combined pool of all distinct individuals with a Personal Health Number (PHN) in the general population of BC and meeting the age eligibility criterion (≥ 19 years) between April 1, 1996 and March 31, 2013. The PHN is a unique identifier that tracks health care system encounters for all BC residents.

### Outcome and definitions

Our primary outcome was death from any cause. Deaths were identified through the BC Vital Statistics Agency mortality dataset [31], which contains information on all registered deaths in BC and was accessed through a data linkage conducted by Population Data BC. As part of the death registration in BC as well as elsewhere in Canada, a medical certificate of cause of death is completed by a medical examiner, coroner or other certifier, and elicits relevant information on the nature of the death. We classified deaths based on the underlying cause of death information into categories by causes using ICD-9 codes for deaths through 1999, and ICD-10 codes for deaths from 2000 onwards. The causes of death were grouped into the following categories: HIV/AIDS-related, cancers, CVD, chronic respiratory diseases, drug abuse and overdose, hepatic and liver diseases, neurologic disorders, renal diseases, suicides, unintentional injuries (including accidents), other infectious and parasitic diseases (excluding HIV and viral hepatitis), and ‘other causes’, which includes all causes of death not listed in the aforementioned categories. Since deaths from cancers such as Kaposi sarcoma, cervical cancer and Non-Hodgkin’s lymphoma are typically regarded as AIDS-defining; we grouped codes for such deaths occurring within the HIV-infected cohort under the ‘HIV/AIDS-related’ cause of death category and excluded them from the ‘cancer’ category. Table 1 lists the ICD-9/10 codes associated with the causes of death categories.

**Table 1:** Causes of death categories and the applicable ICD 9 and 10 codes

Cause of death	ICD 9 code	ICD 10 code
HIV/AIDS-related	042-044	B20-B24
Cancer^a^	140-239	C00-D48
Cardiovascular diseases	410-414, 428, 430-438, 362.3	I00-I99
Chronic respiratory diseases	491, 492, 496, 493	J40-J46
Drug abuse and overdose	304, 305, E850-E858	F10-F19, F55, X40-X45, T40
Hepatic and liver diseases	070, 570-572, 275, 456, 155, 782.4	B15-B19, K70-K77
Neurologic disorders	320-327, 330-359	F03, G00-G99
Renal diseases	250.4, 403-404, 580-590, 593, 792.5, V45.1, V42.0	N00-N07, N17-N19, N25-N27
Suicides	E950-E959	X60-X84, Y87.0
Unintentional injuries (including accidents)^b^	800-999, E800-E999	V01-Y89
Other infectious and parasitic diseases^c^	001-139, 480-487	A00-B99, J10-J18
Other causes	All codes not previously listed	All codes not previously listed

Legend: *ICD* International Classification of Diseases

^a^Since deaths from cancers such as Kaposi sarcoma, cervical cancer and Non-Hodgkin’s lymphoma are typically regarded as AIDS-defining; we grouped codes for such deaths occurring within the HIV-infected cohort under the ‘HIV-related’ cause of death category and excluded them from the ‘cancer’ category. The applicable codes are: 176, 180, 200, 202 (ICD 9) and C46, C53, C82 C83 (ICD 10)

^b^Excludes suicides, drug abuse and overdose codes

^c^Excludes HIV and viral hepatitis codes

### Statistical analysis

Crude all-cause and cause-specific mortality rate per 1000 person-years (PY) were calculated from the observed number of deaths and PYs of observation. To account for differences in the age structure and to permit comparability between the HIV-infected and uninfected cohorts, we computed the age-standardized mortality rates (ASMR) using the direct standardization method with the 1991 Canadian censual population as the reference standard population. The 1991 Canadian population is typically used for the standardization of age-specific disease rates in Canada. Additionally, we compared the ASMRs in the cohorts per calendar interval. We divided the observation period into 11 calendar intervals: 1996, 1997–1998, 1999–2000, 2001–2002, 2003–2004, 2005–2006, 2007–2008, 2009–2010, and 2011–2012). Although shorter than the other calendar periods, we elected to include 1996 (available data from April to December) to enable us capture mortality around the period of cART introduction. The remaining 10 calendar intervals were 2-year periods each. In sensitivity analyses, we investigated trends over time in ASMR among HIV-infected individuals according to antiretroviral drug uptake (ever vs. never), use of triple-drug cART at treatment initiation (yes vs. never), both compared to uninfected individuals. We calculated the mortality rate ratio as an estimate of the relative risk comparing HIV-infected and uninfected individuals by dividing the ASMRs of HIV-infected individuals by that of uninfected individuals. The 95% confidence intervals (CI) for the mortality rates and rate ratios were calculated by Normal approximation assuming a Poisson distribution [33, 34]. Population figures for the Canadian standard population were obtained from Statistics Canada [35]. As a sensitivity analysis for comparison purposes only, we repeated the standardization of the mortality rates using the latest available Canadian censual population (2011) [35].

Comparisons between groups were performed using Chi-square test or Fisher exact test for categorical variables and Kruskall-Wallis test for continuous variables. Test of trend over time were performed using Kendall rank correlation. Statistical significance is defined at a 0.05 level. All data manipulation and statistical analyses were performed using SAS 9.4 (Cary NC, USA) and R Statistical Program, version 3.2.2 (Vienna, Austria).

## Results

### Participant characteristics

From April 1996 to December 2012, 13,729 HIV-infected and 510,313 uninfected individuals – a combined 524,042 persons – contributed a follow-up of 108,990 PYs (median: 7.22 [25^th^, 75^th^ percentile: 2.96, 12.97] years per person) and 5,511,160 PYs (median: 12.70 [4.76, 16.75] years per person) of observation, respectively to the analysis. The baseline characteristics of the study participants are described in Table 2. Compared to uninfected individuals, HIV-infected individuals were more likely to be older at cohort entry (median age: 38 vs 36 years, *p* < 0.001) and more likely to be male (80% vs. 50%, *p* < 0.001). Roughly one-quarter of HIV-infected individuals had no record of HIV treatment initiation. Among those who have ever received ART, approximately 37% initiated therapy with an AIDS-defining CD4 count (< 200 cells/mm^3^).

**Table 2:** Characteristics of study participants

Characteristics	Entire sample, n (%)			Dead, n (%)		
	HIV+ (*N* = 13729)	HIV- (*N* = 510313)	*p*-value	HIV+ (*N* = 3401)	HIV- (*N* = 47647)	*p*-value
Age at study entry, median (Q1, Q3) years	38 (32, 46)	36 (24, 50)	<0.001	41 (34, 49)	71 (59, 78)	<0.001
Age at death, median (Q1, Q3) years				46 (39, 55)	80 (69, 87)	<0.001
Sex						
Male	11017 (80.25)	256440 (50.25)	<0.001	2716 (79.86)	24394 (51.20)	<0.001
Female	2712 (19.75)	253873 (49.75)		685 (20.14)	23253 (48.80)	
Follow-up time, median (Q1, Q3) years	7.22 (2.96, 12.97)	12.70 (4.76,16.75)	<0.001	3.91 (1.16, 7.60)	8.42 (4.25, 12.58)	<0.001
Antiretroviral therapy ever?						
Yes	10165 (74.04)			2377 (69.89)		
No	3564 (25.96)	--		1024 (30.11)	--	

Legend: Q1, 25^th^ percentile; Q3, 75^th^ percentile

### Mortality during follow-up

During the approximately 17 years of observation, a total of 3401 HIV-infected (25%) and 47,647 uninfected individuals (9%) died. Compared to uninfected individuals, HIV-infected individuals were more likely to die young (median age: 46 vs 80 years, *p* < 0.001), with approximately 75% of the deaths occurring before or at 55 years of age compared to 87 years among uninfected individuals (Table 2). Table 3 shows the number of deaths and crude mortality rates by age, sex and cause of death. The proportion of deaths among HIV-infected women (25.3%) was not significantly different from that for infected men (24.7%) (*p* = 0.529). Conversely, the proportion of deaths among uninfected women was lower compared to uninfected men (9.2% vs. 9.5%, *p* < 0.001).

**Table 3:** Frequency of deaths and crude mortality rates by age, sex and causes of death (1996-2012)

	HIV-infected				HIV-uninfected			
	Deaths (%)	*p*-value	PY (per 1,000)	CMR per 1,000 PY (95% CI)	Deaths (%)	*p*-value	PY (per 1,000)	CMR per 1,000 PY (95% CI)
Age at cohort entry (years)								
20–34	873 (17.44)	<0.001	43.31	20.16 (18.82–21.50)	1,929 (0.78)	<0.001	2,321.61	0.83 (0.79–0.87)
35–49	1,723 (25.92)		52.75	32.66 (31.12–34.21)	4,733 (3.50)		1,716.51	2.76 (2.68–2.84)
50–64	582 (34.13)		11.24	51.78 (47.58–55.99)	9,639 (13.52)		916.26	10.52 (10.31–10.73)
65+	223 (60.43)		1.7	131.37 (114.13–148.62)	31,346 (56.48)		556.77	56.30 (55.68–56.92)
Sex								
Male	2,716 (24.65)	0.529	88.76	30.60 (29.45–31.75)	24,394 (9.51)	<0.001	2,704.30	9.02 (8.91–9.13)
Female	685 (25.26)		20.23	33.85 (31.32–36.39)	23,253 (9.16)		2,806.86	8.28 (8.18–8.39)
ARV ever?								
Yes	2,377 (23.38)	<0.001	90.95	26.14 (25.08–27.19)				
No	1,024 (28.73)		18.04	56.75 (53.28–60.23)				
Cause of death								
All-cause	3,401 (24.77)		108.99	31.20 (30.16–32.25)	47,647 (9.34)		5,511.16	8.65 (8.57–8.72)
HIV/AIDS-related	1,698 (12.37)		108.99	15.58 (14.84–16.32)	0 (0.00)		5,511.16	0.00 (0.00–0.00)
Cancers	297 (2.16)		108.99	2.72 (2.42–3.03)	13,950 (2.73)		5,511.16	2.53 (2.49–2.57)
Cardiovascular diseases	238 (1.73)		108.99	2.18 (1.91–2.46)	15,295 (3.00)		5,511.16	2.78 (2.73–2.82)
Chronic respiratory diseases	52 (0.38)		108.99	0.48 (0.35–0.61)	2,179 (0.43)		5,511.16	0.40 (0.38–0.41)
Drug abuse and overdose	380 (2.77)		108.99	3.49 (3.14–3.84)	696 (0.14)		5,511.16	0.13 (0.12–0.14)
Hepatic and liver diseases	150 (1.09)		108.99	1.38 (1.16–1.60)	755 (0.15)		5,511.16	0.14 (0.13–0.15)
Neurologic disorders	19 (0.14)		108.99	0.17 (0.10–0.25)	3,273 (0.64)		5,511.16	0.59 (0.57–0.61)
Renal diseases	21 (0.15)		108.99	0.19 (0.11–0.28)	746 (0.15)		5,511.16	0.14 (0.13–0.15)
Suicides	88 (0.64)		108.99	0.81 (0.64–0.98)	712 (0.14)		5,511.16	0.13 (0.12–0.14)
Unintentional injuries	87 (0.63)		108.99	0.80 (0.63–0.97)	1,792 (0.35)		5,511.16	0.33 (0.31–0.34)
Other infectious and parasitic diseases	101 (0.74)		108.99	0.93 (0.75–1.11)	1,998 (0.39)		5,511.16	0.36 (0.35–0.38)
Other causes	270 (1.97)		108.99	2.48 (2.18–2.77)	6,251 (1.22)		5,511.16	1.13 (1.11–1.16)

Legend: *ARV* Antiretroviral (drug), *CI* Confidence Intervals, *CMR* Crude mortality rate, *PY* Person-years

### Causes of death

**Fig. 1:**
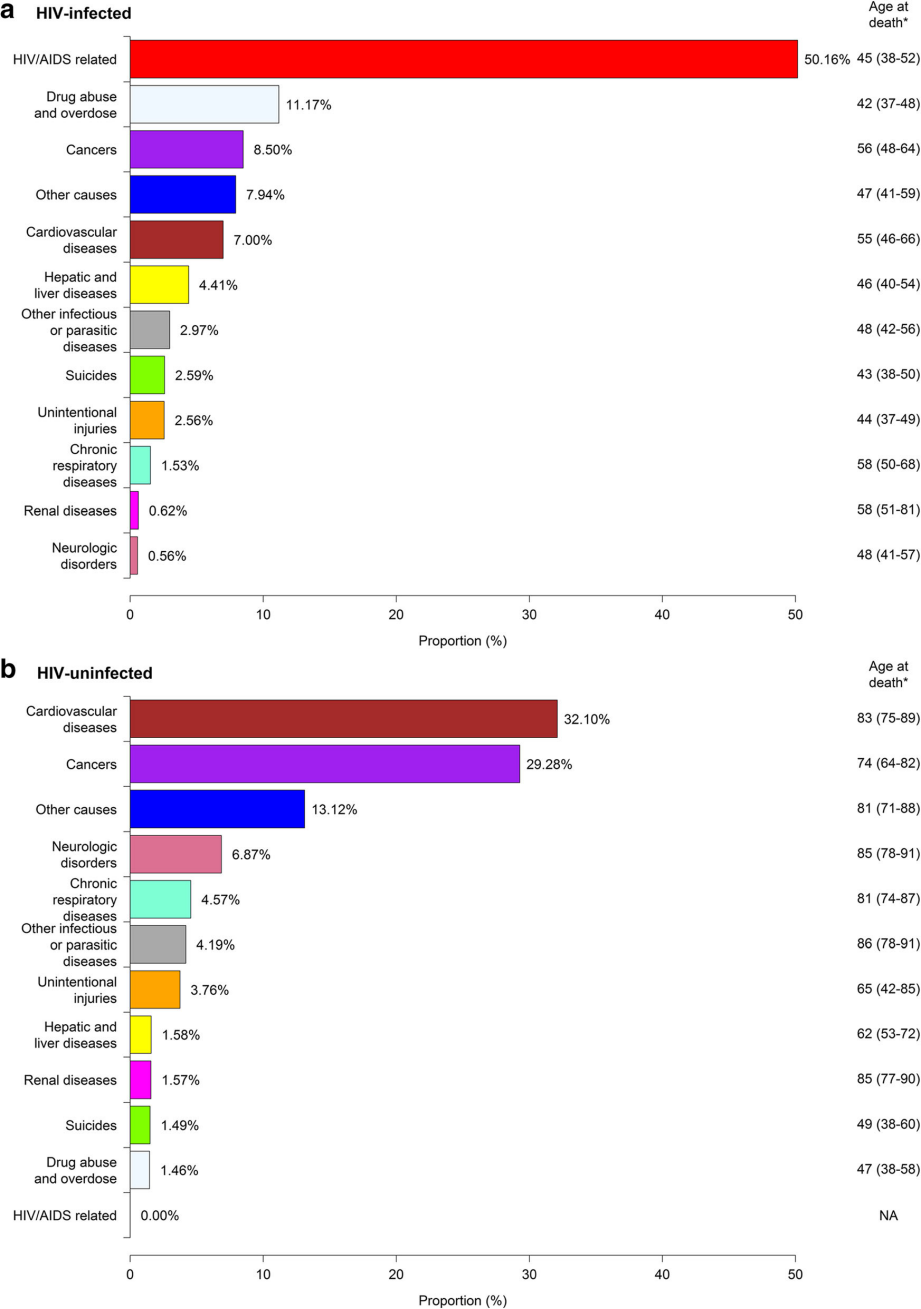
Overall distribution of the proportion of deaths by causes and by HIV status in British Columbia, during 1996-2012. Legend: *, Values are in the format ‘median age (25^th^ percentile-75^th^ percentile)’ years; Note: The ‘cancer’ category in Fig. 1a excludes deaths attributable to Kaposi sarcoma, cervical cancer and Non-Hodgkin’s lymphoma, as these have been accounted for within HIV/AIDS-related deaths. **a** HIV-infected, **b** HIV-uninfected

**Fig. 2:**
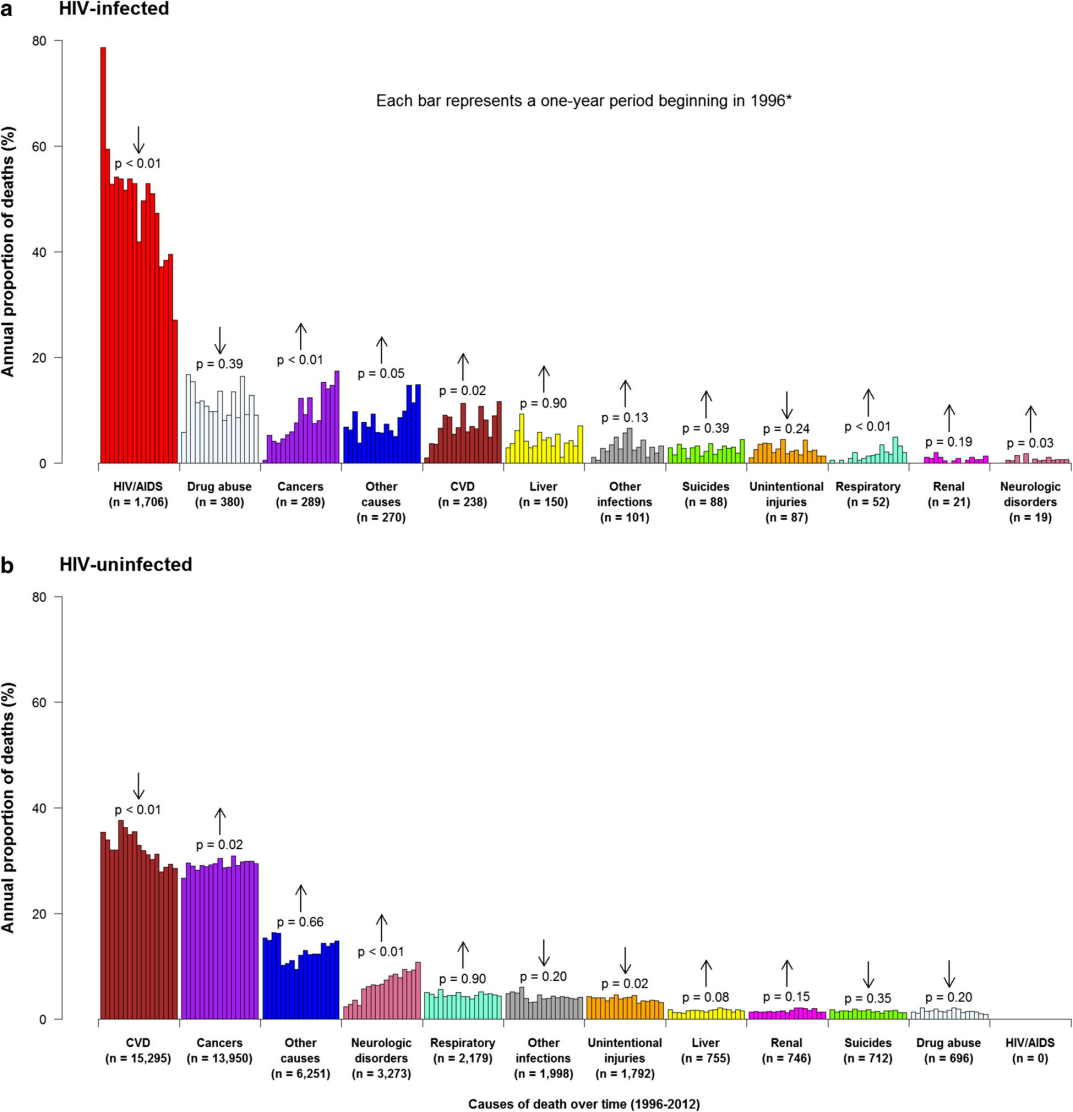
Annual changes in the proportion of deaths by causes and by HIV status in British Columbia, during 1996–2012. Legend: CVD, Cardiovascular diseases; *, Each bar in the figure represents a one-year period beginning in 1996 (except for the first bar – 1996 –, which is nine months long); Note: The ‘cancer’ category in Fig. 2a excludes deaths attributable to Kaposi sarcoma, cervical cancer and Non-Hodgkin’s lymphoma, as these have been accounted for within HIV/AIDS-related deaths. **a** HIV-infrcted, **b** HIV-uninfected

Figures 1 and 2 show the distribution and annual changes in the proportion of deaths across causes among the study participants during the observation period. Although HIV/AIDS-related deaths accounted for 50% of the total deaths among HIV-infected individuals during follow-up (Fig. 1), the proportion of deaths from HIV/AIDS-related causes dropped from 79% in 1996 to 27% by 2012 (*p* < 0.001) (Fig. 2). Overall, the top non-HIV/AIDS-related causes of death during the study follow-up in decreasing order are: drug abuse and overdose (11.2%), cancers (8.5%), CVD (7.0%), and hepatic and liver diseases (4.4%). The majority of deaths classified under ‘other causes’ included deaths from unknown causes and a myriad of other conditions. This cause of death category accounted for 7.9% and 13.1% of mortality among HIV-infected and uninfected individuals respectively.

Among HIV-infected individuals, the proportion of deaths due to HIV/AIDS decreased significantly over time since 1996 (*p* <  0.001), while those from non-HIV/AIDS-related causes either remained stable or increased over time (Fig. 2). In 1996, CVD and non-AIDS-defining cancer accounted for a combined < 2% of deaths among HIV-infected individuals compared to 29% in 2012 (*p* < 0.05). Non-AIDS-defining cancers which accounted for < 1.0% of the deaths in 1996 have recently emerged to become the leading non-HIV/AIDS-related cause of death, accounting for 17% of all deaths among infected individuals by 2012 (*p* < 0.001). Among deaths attributable to non-AIDS defining cancers, lung cancer was the most commonly observed cancer type in both HIV-infected and uninfected individuals. In decreasing order, this was followed by cancer of the liver, colon, pancreas, anus, rectum, stomach and prostate among HIV-infected individuals; and colon, breast, prostate, pancreas, bladder, esophagus, stomach and ovarian cancer among uninfected individuals.

Among uninfected individuals, CVD (32%) and cancers (29%) were the most common accounting for over 60% of the deaths during follow-up. In terms of trend, the proportion of deaths from CVD decreased over time (*p* < 0.001), while deaths due to cancer have increased (*p* = 0.02), overtaking CVD as the leading cause of death in more recent years (Fig. 2). Consequently, in both HIV-infected and uninfected individuals, our results demonstrate that cancers are now the leading non-HIV/AIDS-related cause of death. Compared to HIV-infected individuals where deaths due to drug abuse and overdose was among the leading cause of death, this category accounted for the least proportion of deaths among uninfected individuals during follow-up.

### Changes in age-standardized mortality rate

**Fig. 3:**
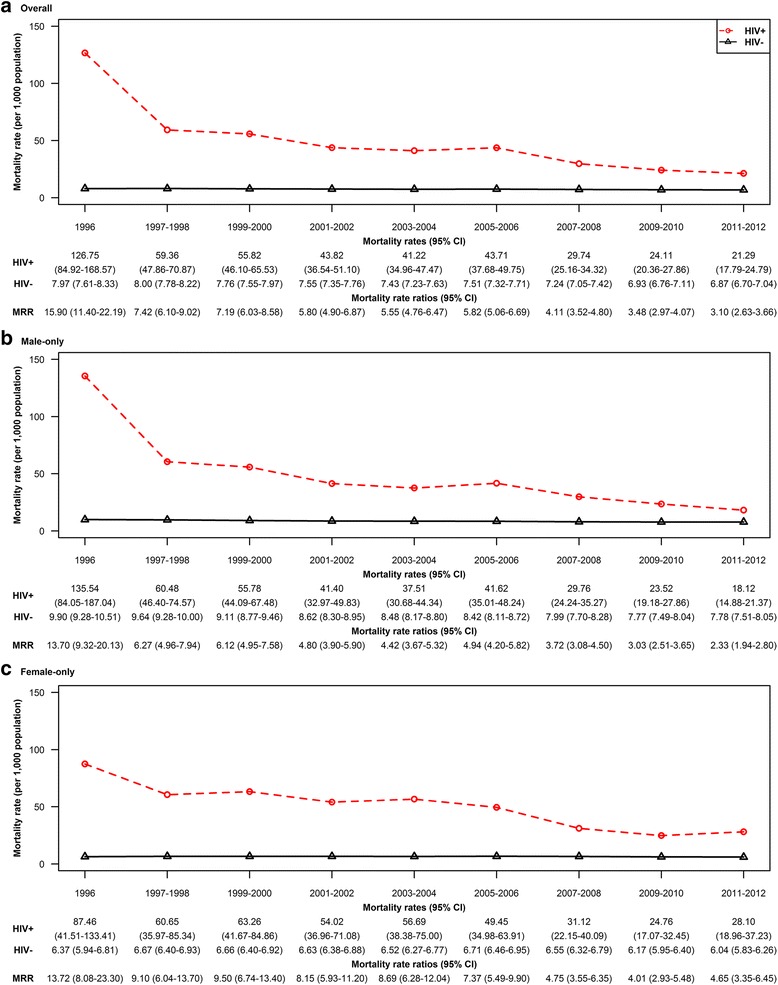
Changes over time in all-cause age-standardized mortality rates, overall and by HIV status and sex in British Columbia, during 1996–2012. Legend: CI, Confidence interval; MRR, Mortality rate ratio. **a** Overall, **b** Male-only, **c** Female only

**Fig. 4:**
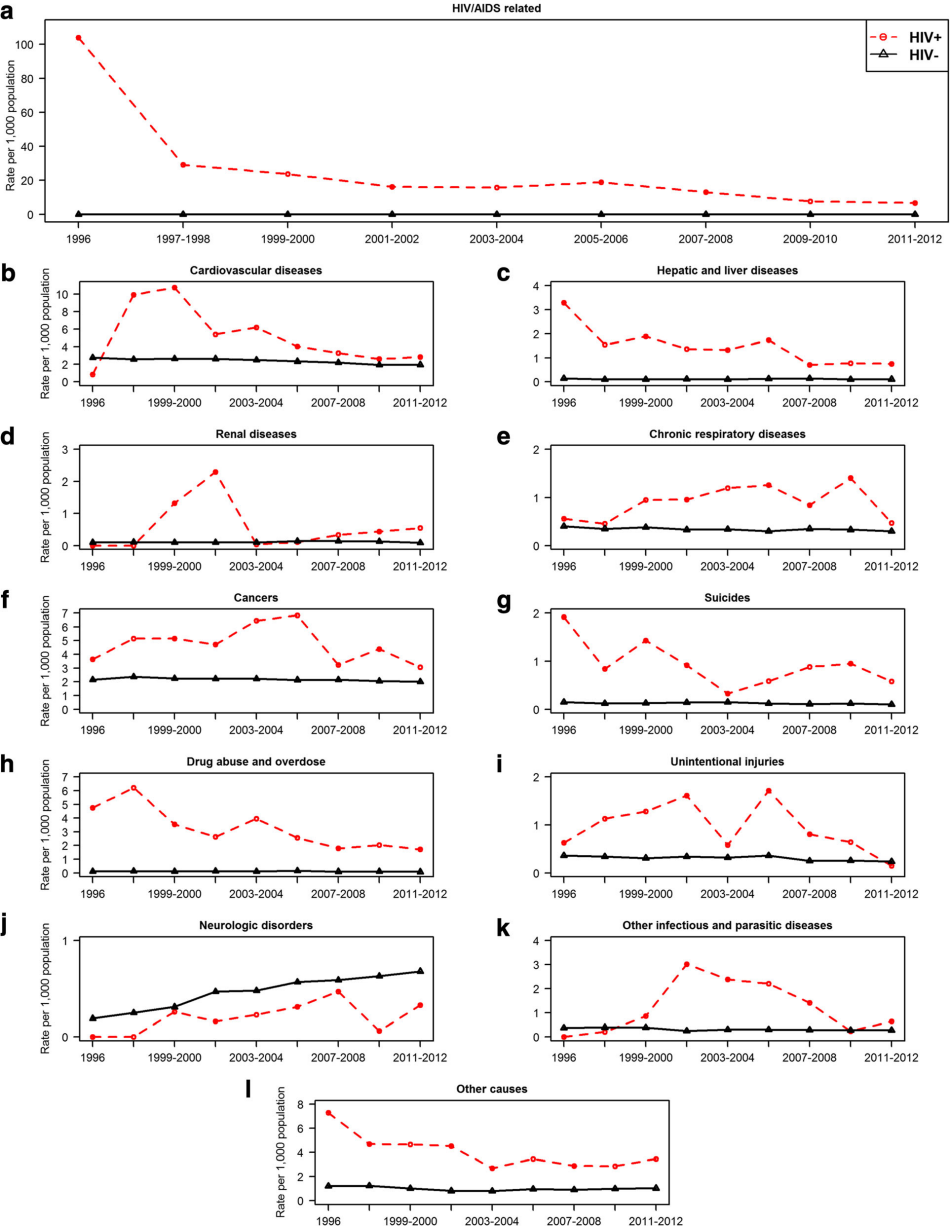
Changes over time in cause-specific age-standardized mortality rates, by HIV status in British Columbia, during 1996–2012. Legend: The ‘cancer’ mortality line graph for HIV-infected individuals (Fig. 4f, red line) excludes deaths attributable to Kaposi sarcoma, cervical cancer and Non-Hodgkin’s lymphoma, as these have been accounted for within HIV/AIDS-related deaths. **a** HIV/AIDS related, **b** cardiovascular diseases, **c** Hepatic and liver diseases, **d** Renal diseases, **e** Chronic respiratory diseases, **f** Cancers, **g** Suicides, **h** Drug abuse and overdose, **i** unintentional injuries, **j** Neurologic disorders, **k** Other infectious and parasitic diseases, **l** Other causes

Figures 3 and 4 describes the changes over time in the age-standardized all-cause and cause-specific mortality rates, by HIV status. Among HIV-infected individuals, the all-cause ASMR decreased from 126.75 (95% CI: 84.92-168.57) per 1000 population in 1996, to 21.29 (95% CI: 17.79-24.79) in 2011-2012 (*p* < 0.001 for trend). In the uninfected population, the all-cause ASMR changed over similar time period, from 7.97 (95% CI: 7.61-8.33) per 1000 population to 6.87 (95% CI: 6.70-7.04), respectively (*p* < 0.001 for trend) (Fig. 3a). This represents an 83% drop in all-cause mortality rate from 1996 to 2011-2012 era among HIV-infected individuals compared to a 14% drop among uninfected individuals. The age-adjusted relative risk of death in HIV-infected compared to uninfected individuals decreased from 15.90 (95% CI: 11.40-22.19) in 1996 to 3.10 (95% CI: 2.63-3.66) in the 2011–2012 era, representing a 81% reduction in the relative risk of mortality over time. When stratified by sex, the relative risk of death comparing infected to uninfected individuals was similar in males and females as of 1996 (13.70 vs 13.72). By the 2011–2012 era, the relative risk of death comparing HIV-infected and uninfected men was 2.33 (95% CI: 1.94-2.80), whereas it was 4.65 (95% CI: 3.35-6.45) among HIV-infected compared to uninfected women (Fig. 3b, 3c).

The ASMR from HIV/AIDS-related deaths decreased significantly from 103.85 in 1996 to 6.72 per 1000 population in the 2011–2012 eras (*p* < 0.001 for trend), representing a 94% reduction in death rate from HIV/AIDS-related causes (Fig. 4a). Over the study’s observation period, significant reductions in mortality rates among HIV-infected individuals were also observed for mortality from hepatic and liver disease, as well as from drug abuse and overdose. Although the proportion of deaths attributable to CVD and non-AIDS-defining cancers significantly increased over time (*p* < 0.05) among HIV-infected individuals (Fig. 2a), the mortality rates from CVD and cancer decreased over time (Fig. 4b, 4f), albeit not significantly (*p* > 0.05). There was a statistically significant increase over time in mortality rates and proportion of deaths attributable to neurological disorders (Figs. 2a and 4j; *p* < 0.05). For chronic respiratory diseases, renal diseases, other infectious and parasitic diseases deaths, mortality rate increases were observed but this was not significant.

Among uninfected individuals, statistically significant mortality rate reductions were observed over time for deaths attributable to CVD, cancers, chronic respiratory diseases, suicides, unintentional injuries and other infectious and parasitic diseases; however mortality rates attributable to neurologic disorders increased significantly over time.

### Sensitivity analyses

**Fig. 5:**
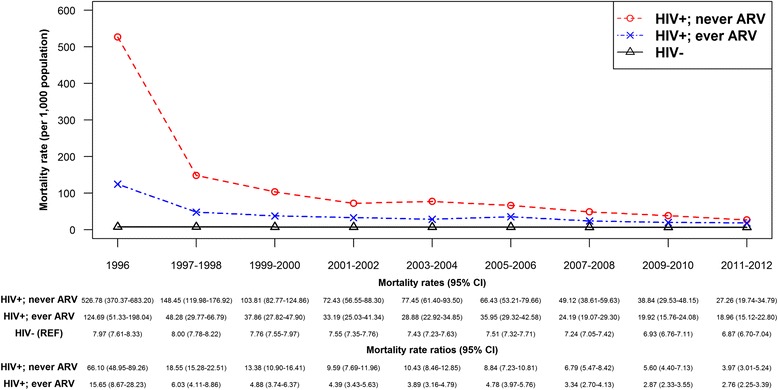
Trend in age-standardized mortality rate, by HIV and antiretroviral therapy uptake status (ever vs. never) in British Columbia, during 1996–2012. Legend: ARV, Antiretroviral (drug); CI, Confidence interval

**Fig. 6:**
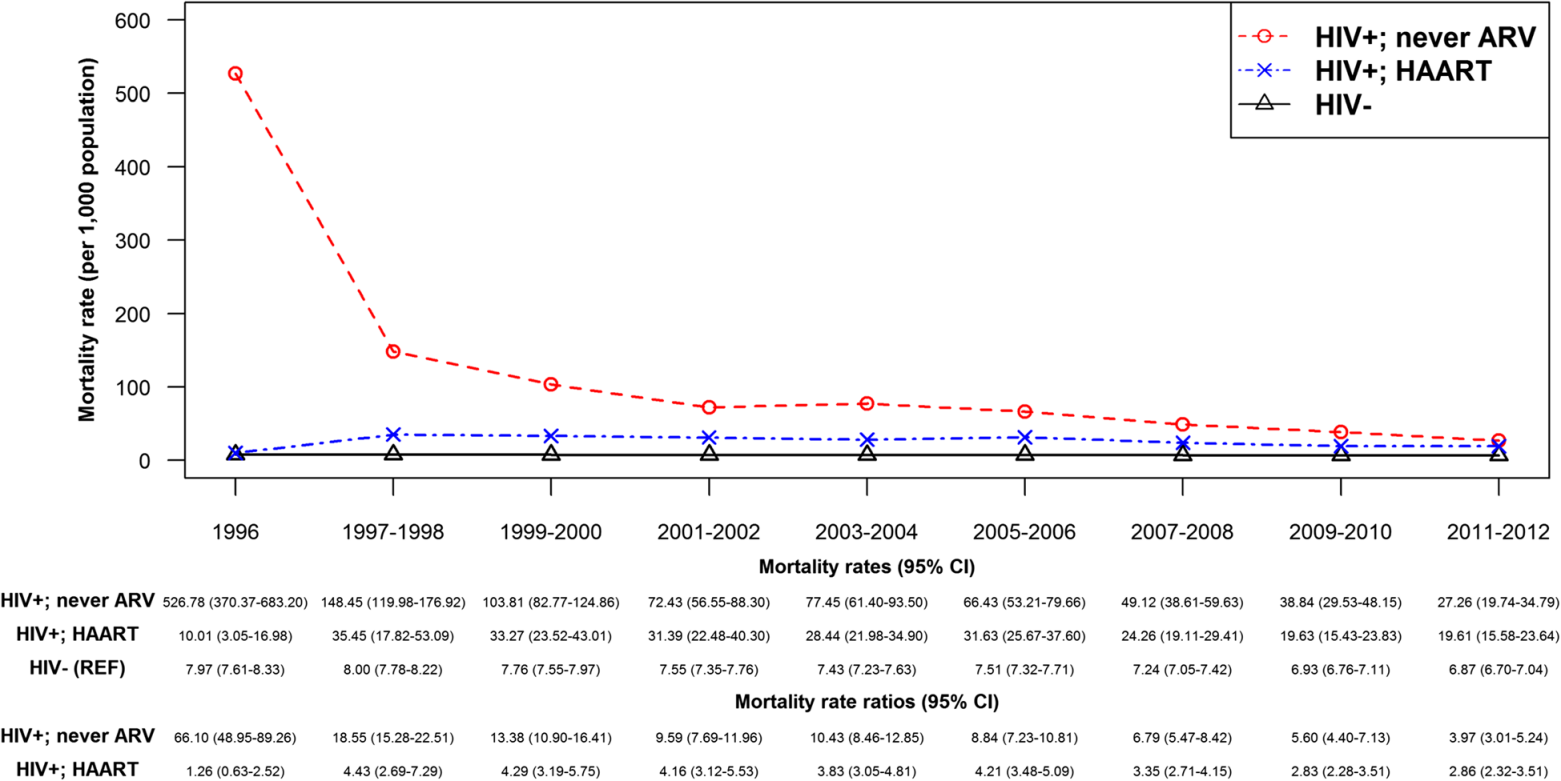
Trend in age-standardized mortality rate, by HIV and highly active antiretroviral therapy (HAART) status at treatment initiation (yes vs. never) in British Columbia, during 1996-2012. Legend: ARV, Antiretroviral (drug); CI, Confidence interval; HAART, Highly active antiretroviral therapy

Although we observed slightly higher ASMRs (data not shown) when we standardized rates using a 2011 Canadian censual population compared to the main analyses that used a 1991 censual population, the overall trends remained unchanged. The slight increase in mortality rates is likely because the 2011 censual population has a higher proportion of individuals in older age groups than the 1991 population. In separate analyses stratified by antiretroviral therapy uptake (ever vs. never) and initiation of therapy with triple-drug cART (yes vs. never on therapy), we observed significant mortality rate reductions over time across all groups (Figs. 5 and 6). During the analytic period, individuals who were never on ART had mortality rates ranging from 1.4 to almost 5 times higher than among those ever on ART or those initiating treatment with triple-drug cART. Of note, 92% of individuals ever on ART in this study initiated therapy on a triple-drug cART.

## Discussion

Since cART introduction in 1996, this is the first population-based study in Canada to characterize changes in mortality and causes of death among persons living with HIV infection as compared to uninfected individuals from the same geographical and health care setting. Compared to uninfected individuals, our result demonstrates that the causes of death among HIV-infected individuals in BC have changed dramatically over time. We observed significant mortality rate reductions in all-cause, HIV/AIDS-related, drug abuse and overdose, and liver disease mortality among people living with HIV in the period from 1996 to 2012. Despite the remarkable decline in mortality from HIV-related causes, HIV/AIDS is still the leading cause of death among HIV-infected individuals and the relative risk of death remains in excess of that for uninfected individuals. Relative to other causes of death, the proportion of total mortality attributable to non-AIDS-defining cancer and CVD has increased among HIV-infected individuals since cART introduction, however, this did not necessarily translate into a trend of increasing mortality rates from these causes; a finding that may be likely due to the overall increased survival in the population. In both HIV-infected and uninfected individuals, we observed significant increases over time in mortality rates from neurologic disorders.

The overall proportion of deaths due to HIV/AIDS-related causes in our study (50.2%) was comparable to those reported from Spain (40.4%) [36], Italy (41.4%) [16], Denmark (44.4%) [37], England and Wales (46.0%) [38], France (47.3%) [39], USA (51.4%) [15] and from multinational cohort collaborations such as Antiretroviral Therapy Cohort Collaboration (49.6%) [14], but higher than that reported in the Data collection on Adverse events of Anti-HIV Drugs [D:A:D] study (28.7%) [40]. While our overall findings are broadly consistent with the mortality declines and cause of death trends presented in previous studies of HIV-infected individuals from other settings [13–16, 38, 40, 41]; variations in the range of such estimates may exist across studies and are likely due to heterogeneity in the population (e.g., by age, sex, CD4 levels, cART history, underlying clinical characteristics, ethnicity), differences in the observation period, or the coding scheme for causes of death categories. In sensitivity analysis stratified by ART uptake (ever vs. never), we observed a better mortality prognosis among those who had ever been treated with ART, thus demonstrating the benefit associated with ART. This is supported by a recent BC study among HIV-infected individuals receiving cART since 2001 which demonstrated that cART initiation was independently associated with reduced mortality [21].

The current study reflects major changes since the introduction of cART in terms of non-HIV/AIDS-related causes; in 1996, only 21% of the deaths among HIV-infected individuals were attributable, as compared to 73% by 2012. Our results confirm two recent reports suggesting that non-AIDS-defining cancers are currently the leading non-HIV/AIDS-related cause of death among HIV-infected individuals [40, 42]. Similarly, we observed that cancers have also recently overtaken CVD to become the principal cause of death among the uninfected population. Like the D:A:D study [40] but unlike a report from the HIV Outpatient Study [15], we observed a statistically significant mortality rate decrease from hepatic and liver disease deaths. Contrary to a finding among HIV-infected hemophiliacs in Canada [43], liver disease is an important cause of death especially among individuals co-infected with HIV and hepatitis C virus [44], although not the main non-HIV/AIDS-related cause of death in the current study. The observed increase in mortality rates attributable to neurologic disorders is likely a reflection of the increased longevity in both populations.

Our results indicating that HIV-infected individuals continue to have a mortality risk ratio that is decreasing as time passes but remains approximately three times more than in uninfected individuals as of 2011-2012 is supported by comparable findings from other studies that have compared mortality rates among persons with and without HIV infection [37, 45]. We suspect the continuing high relative risk of death among HIV-infected, compared to uninfected individuals may in part be explained by the higher prevalence of behavioral risk factors such as smoking, alcohol abuse and other lifestyle factors that are more common among HIV-infected individuals and predisposes them to higher mortality risk compared to uninfected individuals.

Readers should interpret our findings in light of the potential strengths and limitations of the study. The HIV-infected cohort includes the vast majority of all known HIV-infected adults in BC, irrespective of whether they had ever received cART. Despite universal access to HIV treatment in BC, approximately 25% of HIV-infected individuals had no record of HIV treatment initiation during this study (1996 to 2012). Reassuringly, the rates have declined substantially with the implementation of the Treatment as Prevention strategy in BC [46–48], as in a 2008 study we found that 40% of those who died from HIV did not access treatment [49]. Our uninfected cohort includes randomly sampled BC residents who accessed the same universal health care system as the HIV-infected participants, thus minimizing potential selection bias that may arise from using an external comparison group. Unlike most clinic-based cohorts [11, 13, 16, 36, 39, 40, 50], the relatively large, linked and population-based nature of the COAST study is unique to BC and has allowed us to ascertain mortality and cause of death information for all study participants through the same source – the BC Vital Statistics Agency -[31], which is the provincial office that collects vital statistic records in BC. However, since this dataset does not capture deaths to BC residents that occur out-of-province, such deaths were unaccounted for in our analyses. While we suspect this number is likely small, it also will have affected the HIV-infected and uninfected cohorts equally. Finally, our cause of death assessment is limited by the accuracy of the ICD-9/10 coding used for attribution of the underlying cause of death; and the study as a whole, by unmeasured confounding due to its observational nature.

## Conclusion

To sum up, our results suggest that significant improvements have been made in reducing mortality over time from HIV/AIDS-related and several non-HIV/AIDS-related causes of death among people living with HIV. Despite this progress, HIV-infected individuals still have excess mortality risk compared to their uninfected counterparts. Additional efforts are needed to promote effective risk factor management and appropriate screening measures among people living with HIV. Ongoing monitoring of mortality trends by causes will be important and will provide the necessary data to enable us optimally target our efforts at the conditions that influence morbidity and mortality outcomes. Ultimately, in both HIV-infected and uninfected populations, the management of cancers and CVD as well as their known risk factors will be key in improving overall survival outcomes.

## Abbreviations

ART: Antiretroviral therapy
ARV: Antiretroviral (drug)
ASMR: Age-standardized mortality rate
BC: British Columbia
BCCfE: BC Centre for Excellence in HIV/AIDS
cART: Combination antiretroviral therapy
CI: Confidence interval
COAST: Comparative Outcomes And Service Utilization Trends study
CVD: Cardiovascular disease
D:A:D: Data collection on Adverse events of Anti-HIV Drugs study
ICD: International Classification of Diseases
MRR: Mortality rate ratio
PY: Person-years
